# Genome assembly of the snow lotus species *Saussurea involucrata* provides insights into acacetin and rutin biosynthesis and tolerance to an alpine environment

**DOI:** 10.1093/hr/uhad180

**Published:** 2023-09-05

**Authors:** Yanxia Sun, Aidi Zhang, Jacob B Landis, Wei Shi, Xiujun Zhang, Hang Sun, Hengchang Wang

**Affiliations:** CAS Key Laboratory of Plant Germplasm Enhancement and Specialty Agriculture, Wuhan Botanical Garden, Chinese Academy of Sciences, Wuhan 430074, Hubei, China; Center of Conservation Biology, Core Botanical Gardens, Chinese Academy of Sciences, Wuhan 430074, Hubei, China; CAS Key Laboratory of Plant Germplasm Enhancement and Specialty Agriculture, Wuhan Botanical Garden, Chinese Academy of Sciences, Wuhan 430074, Hubei, China; Center of Economic Botany, Core Botanical Gardens, Chinese Academy of Sciences, Wuhan 430074, Hubei, China; BTI Computational Biology Center, Boyce Thompson Institute, Ithaca, NY 14853, USA; School of Integrative Plant Science, Section of Plant Biology and the L.H. Bailey Hortorium, Cornell University, Ithaca, NY 14853, USA; Xinjiang Institute of Ecology and Geography, Chinese Academy of Sciences, Urumchi 830011, Xinjiang, China; CAS Key Laboratory of Plant Germplasm Enhancement and Specialty Agriculture, Wuhan Botanical Garden, Chinese Academy of Sciences, Wuhan 430074, Hubei, China; Center of Economic Botany, Core Botanical Gardens, Chinese Academy of Sciences, Wuhan 430074, Hubei, China; Key Laboratory for Plant Diversity and Biogeography of East Asia, Kunming Institute of Botany, Chinese Academy of Sciences, Kunming 650201, Yunnan, China; CAS Key Laboratory of Plant Germplasm Enhancement and Specialty Agriculture, Wuhan Botanical Garden, Chinese Academy of Sciences, Wuhan 430074, Hubei, China; Center of Conservation Biology, Core Botanical Gardens, Chinese Academy of Sciences, Wuhan 430074, Hubei, China

Dear Editor,

Understanding the evolution and survival mechanisms of endangered wild medicinal herbs is crucial for their cultivation, utilization, and conservation. The snow lotus species *Saussurea involucrata* (Kar. & Kir.) Sch. Bip. (2n = 32) (i.e. the well-known Tianshan snow lotus) which belongs to the eudicot family Asteraceae, is a famous traditional Chinese medicinal herb having anti-inflammatory, antioxidant, and anti-cancer effects; the major bioactive components that exhibit clinical functions in this plant are acacetin, hispidulin, and rutin [1]. *S. involucrata* grows in rock fissures (Fig. 1A) with elevations of 2400–4100 m in the Tianshan and Altai Mountains, surviving in harsh alpine environments characterized by low temperatures and strong ultraviolet radiation. The growth rate is slow, taking 6–8 years for *S. involucrata* to go from seed germination to flowering. Due to the distinct habitat, the resources of *S. involucrata* are rather rare and the species has fallen into endangered status in China due to over-collecting [2].

**Figure 1:**
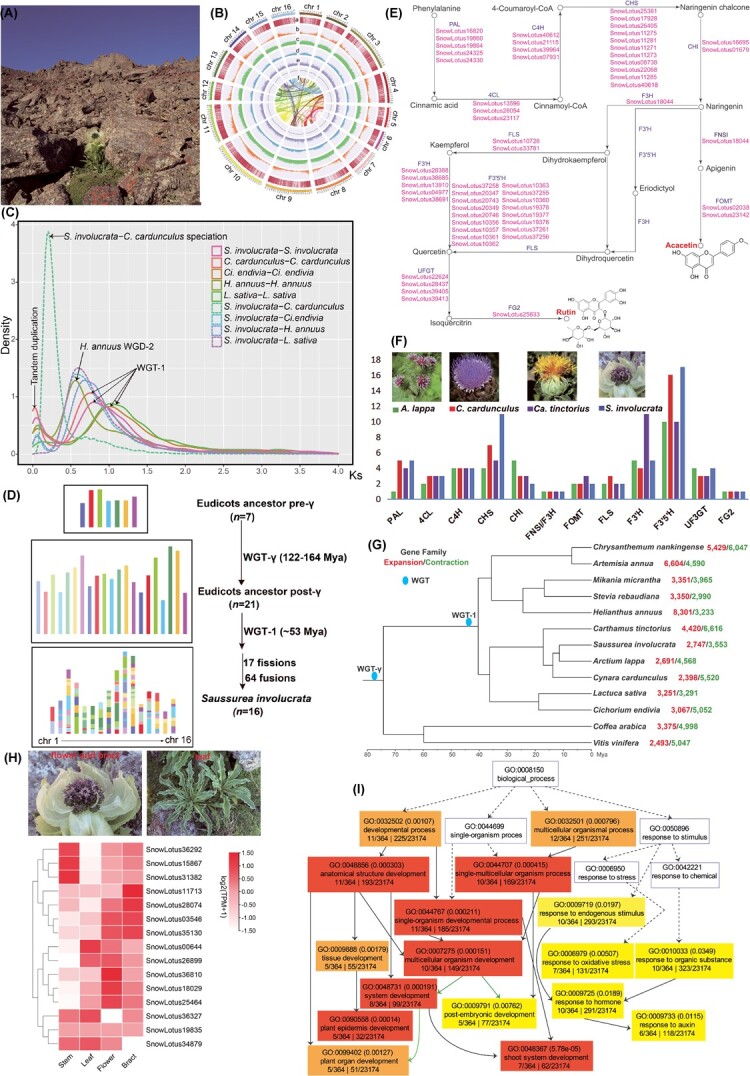
Assembly, annotation, and analyses of the *Saussurea involucrata* genome*.* All *S. involucrata* photographs are taken by Dr. Bing Liu from the Institute of Botany, Chinese Academy of Sciences. (**A**) Photographs showing habitat of *S. involucrata.* (**B**) Genomic landscape of *S. involucrata.* (f) GC content, (e) Gypsy LTR density, (d) Copia LTR density, (c) total LTR density, (b) gene density, and (a) gene expression in leaf. (**C**) Distribution of synonymous substitution (*Ks*) of *S. involucrata* paralogues, *Cynara cardunculus* paralogues, *Cichorium endivia* paralogues, *Helianthus annuus* paralogues, *Lactuca sativa* paralogues, *S. involucrata-C. cardunculus* orthologues, *S. involucrata-Ci. endivia* orthologues, *S. involucrata-H. annuus* orthologues, and *S. involucrata-L. sativa* orthologues. (**D**) Evolutionary scenario of *S. involucrata* from the ancestral eudicot karyotype. (**E**) Predicted candidate genes involved in acacetin and rutin biosynthesis in *S. involucrata.* (**F**) Comparison of gene numbers in gene families involved in acacetin and rutin biosynthesis between *S. involucrata* and three other Asteraceae species. The images of *C. cardunculus*, *Arctium lappa,* and *Carthamus tinctorius* are from Plant Photo Bank of China (PPBC). (**G**) Phylogenetic analysis among 13 eudicot species, including *S. involucrata* and 10 other Asteraceae species, with information of expansion/contraction of gene families. (**H**) Expression profiles of identified genes related to cold resistance in *S. involucrata* across four tissues: stem, leaf, flower, and bract. (**I**) Enriched GO terms (highlighted with colors) of genes specifically expressed in *S. involucrata* bracts.

In the current study, we developed a chromosome-scale genome assembly for *S. involucrata* (accession BGD2108) by combing PacBio HiFi sequencing and High-throughput chromosome conformation capture (Hi-C) technology. Using HiFiasm [3], we obtained 338 high quality contigs (contig N50 = 90 Mb) with a total assembly size of 2452 Mb (Table 1). In total, we placed 94.7% of the contigs on 16 pseudochromosomes (Table 1; Fig. 1B). The completeness of the *S. involucrata* genome assembly evaluated by Benchmarking Universal Single Copy Orthologs (BUSCO) [4] showed 98.7% complete matches in the Embryophyta version 10 dataset (Table 1). The *S. involucrata* genome assembly assessed by Merqury [5] revealed a consensus quality value (QV) of 51.08 (corresponding to a base accuracy of 99.999%) and a completeness rate of 97.23%. Collectively, the above results indicate that the *S. involucrata* genome assembly is of high quality.

The *S. involucrata* genome assembly contains 82.77% repetitive sequences (2030 Mb), of which long terminal repeats (LTRs) accounted for the largest percentage (40.43%) (Table S1, see online supplementary material). We predicted a total of 44 486 protein-coding genes in the *S. involucrata* genome assembly, among which 42 580 (95.7%) were functionally annotated (Table 1; Table S2, see online supplementary material).

To infer the evolutionary history of *S. involucrata*, we performed a genomic comparison of *S. involucrata* with *Cynara cardunculus* (artichoke), *Cichorium endivia* (curly endive), *Lactuca sativa* (lettuce), and *Helianthus annuus* (sunflower) as representatives of Asteraceae. The ancestral eudicot karyotype (AEK) consists of 7 (pre-γ AEK) or 21 (post-γ AEK) protochromosomes (γ, i.e. WGT-->-γ, indicates the ancestral whole-genome triplication of the Eudicots) [6, 7]. The distribution of synonymous substitutions per synonymous site (*Ks*) between collinear gene pairs revealed that *S. involucrata* only experienced a whole-genome triplication (WGT-1, ~53 mya) event after the WGT-γ event (Fig. 1C and D), which is the same as found in artichoke and lettuce [6, 8]. The syntenic pattern between genomic regions in artichoke and *S. involucrata* (Figs S1 and S2, see online supplementary material) suggested that at least 64 chromosome fusions and 17 chromosome fissions were necessary for *S. involucrata* to reach the modern structure of 16 chromosomes (Fig. 1D).

Acacetin and rutin are two predominant bioactive constituents found within *S. involucrata* [1]. By comparing with homologous genes in *Arabidopsis thaliana*, we predicted *S. involucrata* genes encoding each of 13 enzymes in the acacetin and rutin biosynthetic pathway (Fig. 1E): phenylalanine ammonia-lyase (PAL), cinnamate-4-hydroxylase (C4H), 4-coumarate CoA ligase (4CL), chalcone synthase (CHS), chalcone isomerase (CHI), flavanone-3-hydroxylase (F3H), flavanone-3′-hydroxylase (F3’H), flavanone-3′,5′-hydroxylase (F3’5’H), flavonol synthase (FLS), flavonol 3-O-glucosyltransferase (UF3GT), flavonol-3-O-glucoside L-rhamnosyltransferase (FG2), flavone synthase I (FNSI), and flavonoid O-methyltransferase (FOMT). Overall, we identified 57 candidate genes involved in acacetin and rutin biosynthesis in *S. involucrata* (Fig. 1E). Compared with *C. cardunculus*, *Arctium lappa* and *Carthamus tinctorius*, the number of CHS homologs in *S. involucrata* was increased significantly (Fig. 1F)*.*

The phylogenetic tree using 280 single-copy orthologous genes constructed for *S. involucrata* and ten other Asteraceae species with *Vitis vinifera* and *Coffea arabica* as outgroups revealed the 11 Asteraceae species were clustered into four clades and *S. involucrata* was close to *A. lappa* (Fig. 1G). A total of 38 086 gene families were shared by above 13 species. The number of contracted and expanded gene families in *S. involucrata* were 3553 and 2747, respectively (Fig. 1G). Gene ontology analyses showed that the gene families expanded in *S. involucrata* are enriched in genes related to DNA integration, recombination, replication, and repair (Fig. S3, see online supplementary material) which are fundamental molecular mechanisms and reveal a potential survival strategy of *S. involucrata* under severe abiotic stress conditions. Additionally, we identified 539 gene families specific to *S. involucrata* and found these gene families showed enrichment for genes regulating activity of cysteine-type peptidases (Fig. S4, see online supplementary material) which play significant roles in defense responses against environmental stresses including cold and oxidative stress [9].

**Table 1:** Statistics of the *Saussurea involucrata* genome assembly and annotation

	***Saussurea involucrata***
**Assembly**	
Number of contigs	338
Contig N50 (Mb)	90
Genome length (Mb)	2452
Anchor rate (%)	94.7
BUSCO completeness (%)	98.7
QV by Mercury	51.08
Completeness rate by Mercury (%)	97.23
GC content (%)	38.58
**Annotation**	
Repetitive sequences (%)	82.77
Predicted gene models	44 486
Total functionally annotated	42 580
Mean exon length (bp)	275.46
Number of annotated mRNAs	44 486
Mean mRNA length (bp)	3387.64

*S. involucrata* is known to have great tolerance to cold stress. By conducting comparative analysis with reported genes functionally related to cold resistance, we identified 15 homologous genes in *S. involucrata* from protein families, including transcription factor ICE1, calcium-dependent protein kinase 1, dehydrin, stearoyl-acyl-carrier-protein desaturase, fructose-bisphosphate aldolase, late embryogenesis abundant protein, and cold-regulated 413 plasma membrane protein (Table S3, see online supplementary material). We evaluated the expression profiles of these cold stress-resistance genes across four *S. involucrata* tissues (i.e. stem, leaf, flower, and bract) and found most are highly expressed in flowers and bracts (Fig. 1H).

One striking character of *S. involucrata* is the inflorescences surrounded by well-developed membranous bracts (Fig. 1H). Based on the transcripts per million (TPM) values of the Illumina sequencing data, gene expression levels in *S. involucrata* stem, leaf, flower, and bract were evaluated. Finally, we identified 364 genes uniquely expressed in the bracts of *S. involucrata.* These specifically expressed genes are enriched for genes associated with tissue development, and response to auxin and oxidative stress (Fig. 1I), indicating the bracts may play a critical role for *S. involucrata* to survive under oxidative stress induced by strong ultraviolet light.

In summary, we present a high-quality assembly of *S. involucrata* genome, which will be a great resource for the study of this famous traditional Chinese medicinal herb. The results in the present study lay the foundation for future research on the genes related to acacetin and rutin biosynthesis in *S. involucrata* and provide new insights into understanding the genome evolution and molecular mechanisms underlying abiotic stress tolerance of this species.

## Acknowledgements

This work was supported by grants from the National Natural Science Foundation of China (U2003122) and the Second Tibetan Plateau Scientific Expedition and Research (STEP) program (2019QZKK0502). Sequencing service was provided by Bioyi Biotechnology Co., Ltd. Wuhan, China

## Author contributions

H.W., H.S., and Y.S. developed the idea and designed the experiment; W.S. collected the plant materials; Y.S., A.Z., and X.Z. performed the statistical analyses; Y.S. and J.B.L. interpreted the results and wrote the manuscript. All authors read, edited, and approved the final manuscript.

## Data availability

The raw DNA sequencing reads and the assembled genome of *Saussurea involucrata* have been submitted to NCBI. The BioProject ID is PRJNA991078, the BioSample ID is SAMN36288184.

## Conflict of interest statement

The authors declare no conflicts of interest.

## Supplementary data

Supplementary data is available at *Horticulture Research* online.

## Supplementary Material

Web_Material_uhad180
