# Supplementary material for: Genome assembly of the snow lotus species Saussurea involucrata provides insights into acacetin and rutin biosynthesis and tolerance to an alpine environment
Source: Hortic Res. 2023 Sep 5;10(10):uhad180. doi: 10.1093/hr/uhad180 (PMC10599237; doi:10.1093/hr/uhad180)
Supplement: Web_Material_uhad180 [file web_material_uhad180.zip › Figures and Tables only for review.pdf]

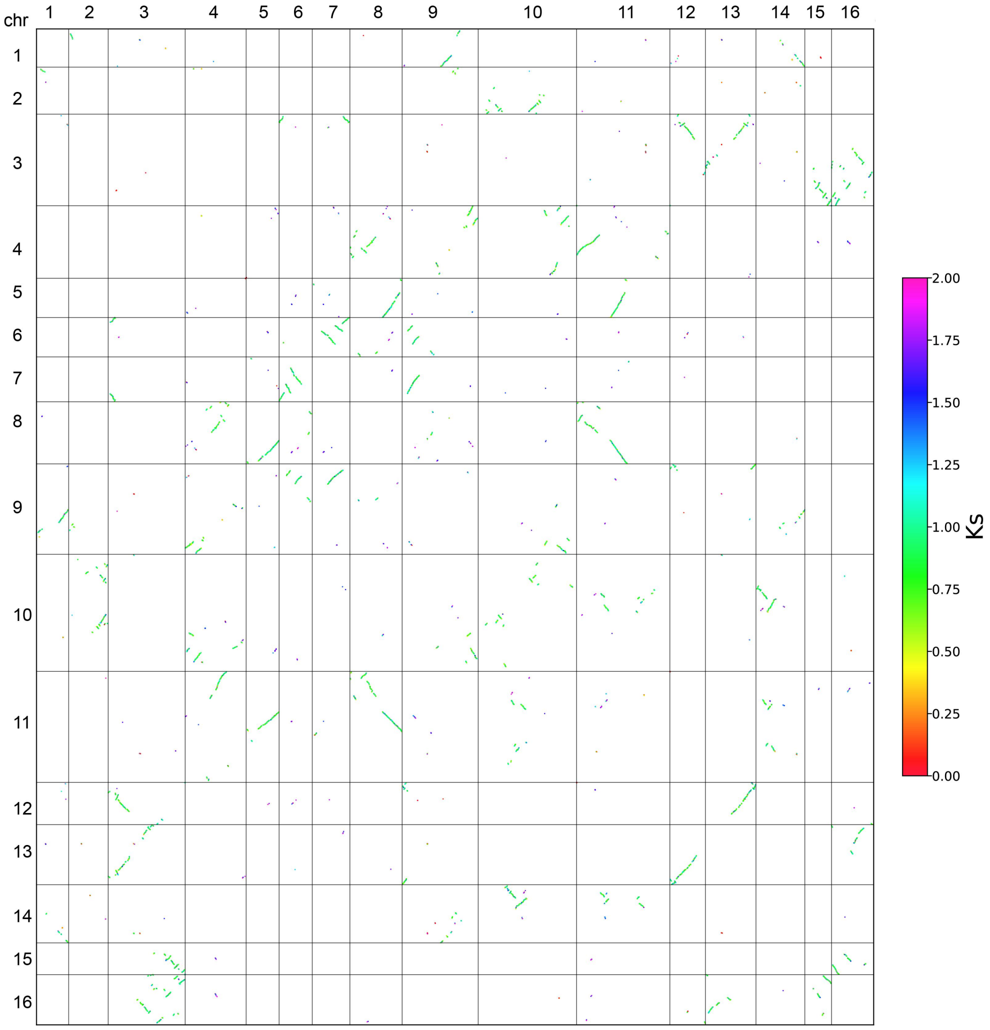

Figure R1. Dot plot showing the DNA sequence alignment of 16 chromosomes in *S. involucrata.*

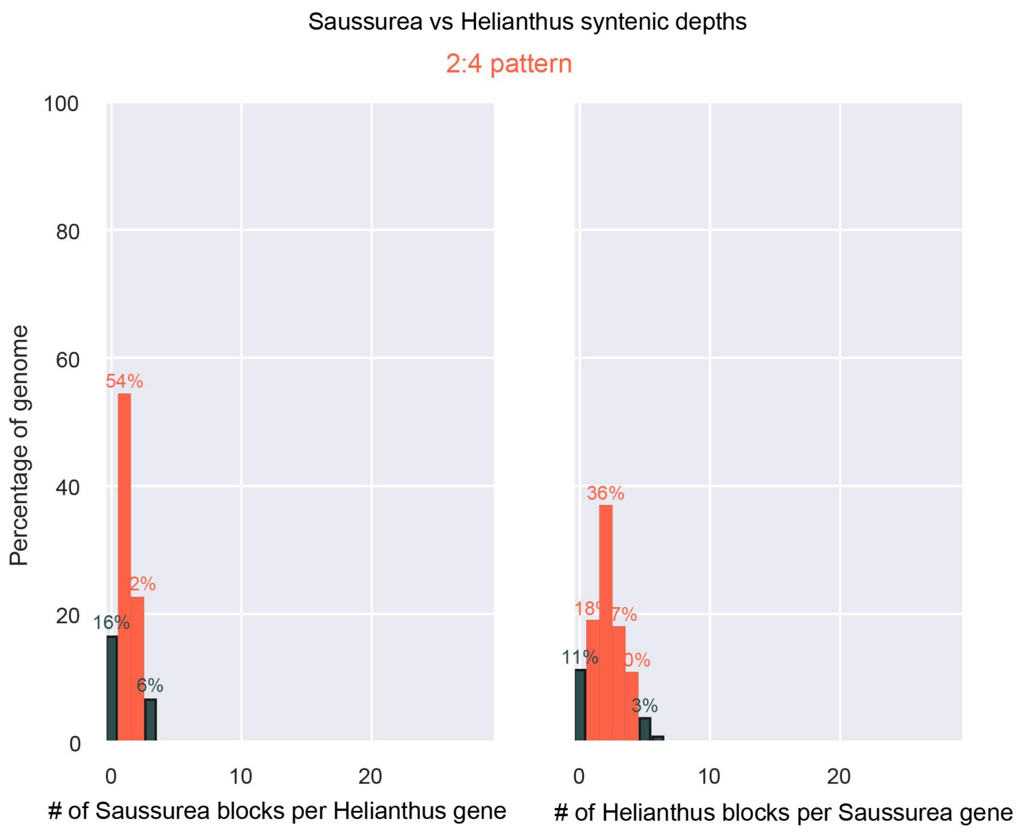

Figure R2. Syntenic depth ratio between *Saussurea involucrata* and *Helianthus annuus*.

Table R1 Comparison of repetitive sequences among different Asteraceae species. SINEs, short interspersed nuclear elements; LINEs, long interspersed nuclear elements.

Species	SINEs (Mb)	LINEs (Mb)	DNA elements (Mb)	LTR elements (Mb)	Unclassified (Mb)	Total (Mb)	Percent
Saussurea involucrata	0	13.63	89.45	991.33	889.11	1983.52	82.77%
Mikania micrantha	5.88	18.98	99.34	813.83	375.5	1,313.53	72.97%
Helianthus annuus	2.21	58.81	99.68	1533.56	549.09	2,243.36	74.78%
Lactuca sativa	1.92	35.13	54.15	1202.08	444.13	1,737.41	72.39%
Artemisia annua	1.1	30.52	58.05	524.54	378.57	992.78	55.15%
Cynara cardunculus	1.27	11.38	37.22	190.69	175.62	416.18	57.39%
Chrysanthemum nankingense	0.68	62.83	81.24	1,128.74	485.28	1,758.77	69.52%
