# Supplementary figures and images for: Genome assembly of the snow lotus species Saussurea involucrata provides insights into acacetin and rutin biosynthesis and tolerance to an alpine environment
Source: Hortic Res. 2023 Sep 5;10(10):uhad180. doi: 10.1093/hr/uhad180 (PMC10599237; doi:10.1093/hr/uhad180)

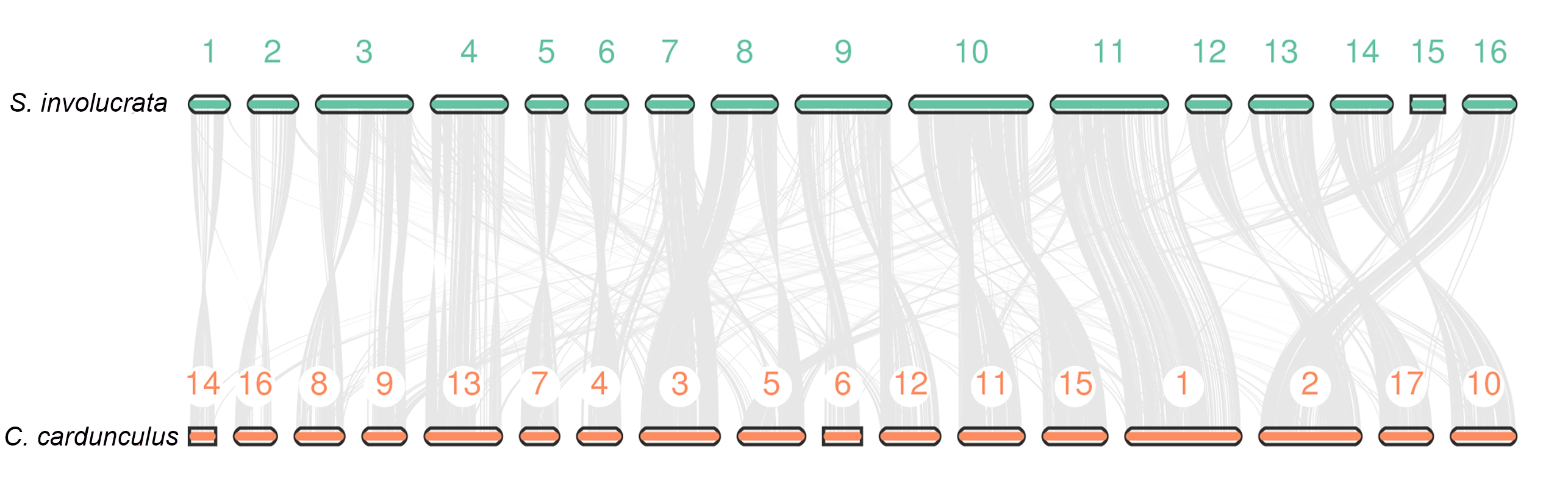

Supplement: Web_Material_uhad180 [file web_material_uhad180.zip › Fig. S1.tif]

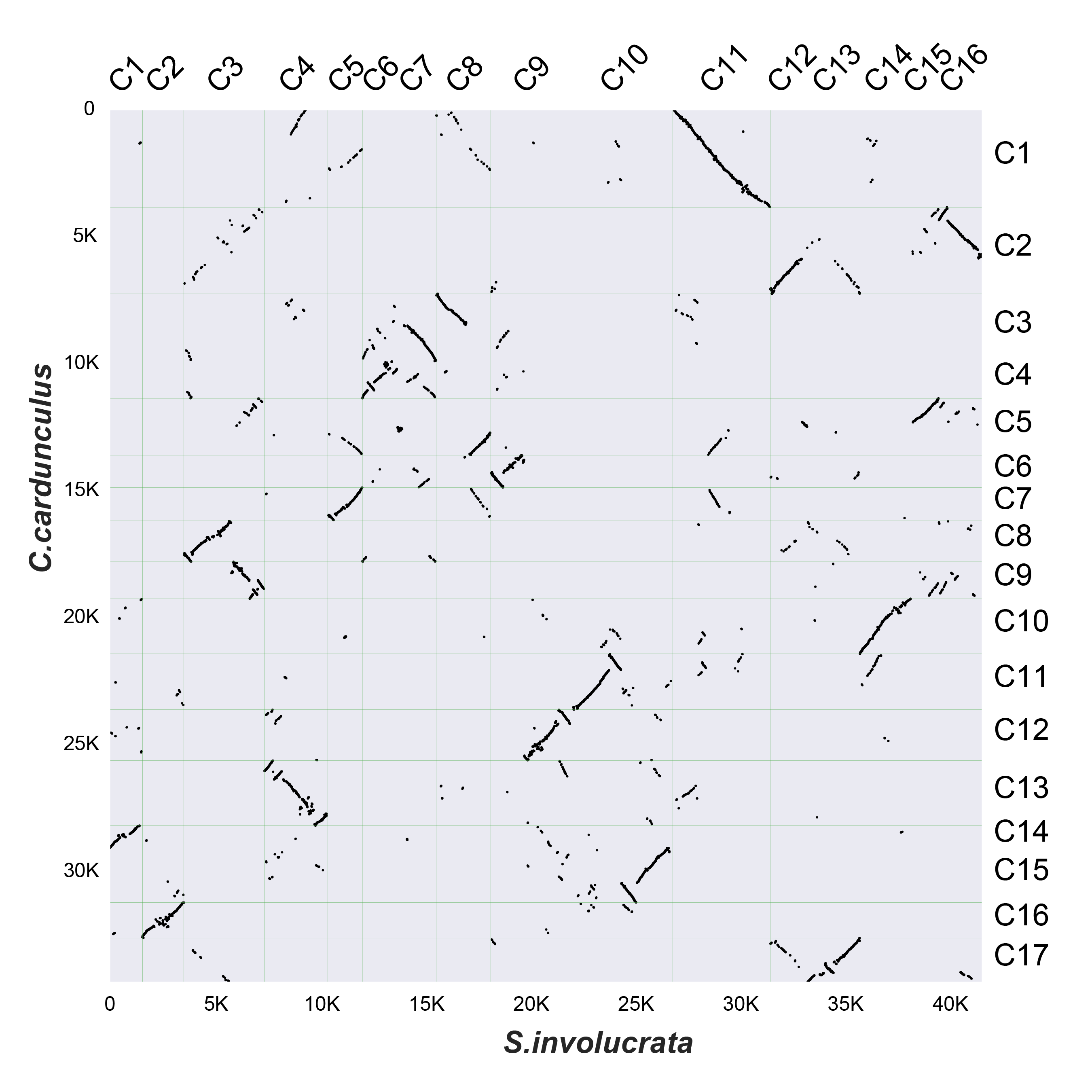

Supplement: Web_Material_uhad180 [file web_material_uhad180.zip › Fig. S2.tif]

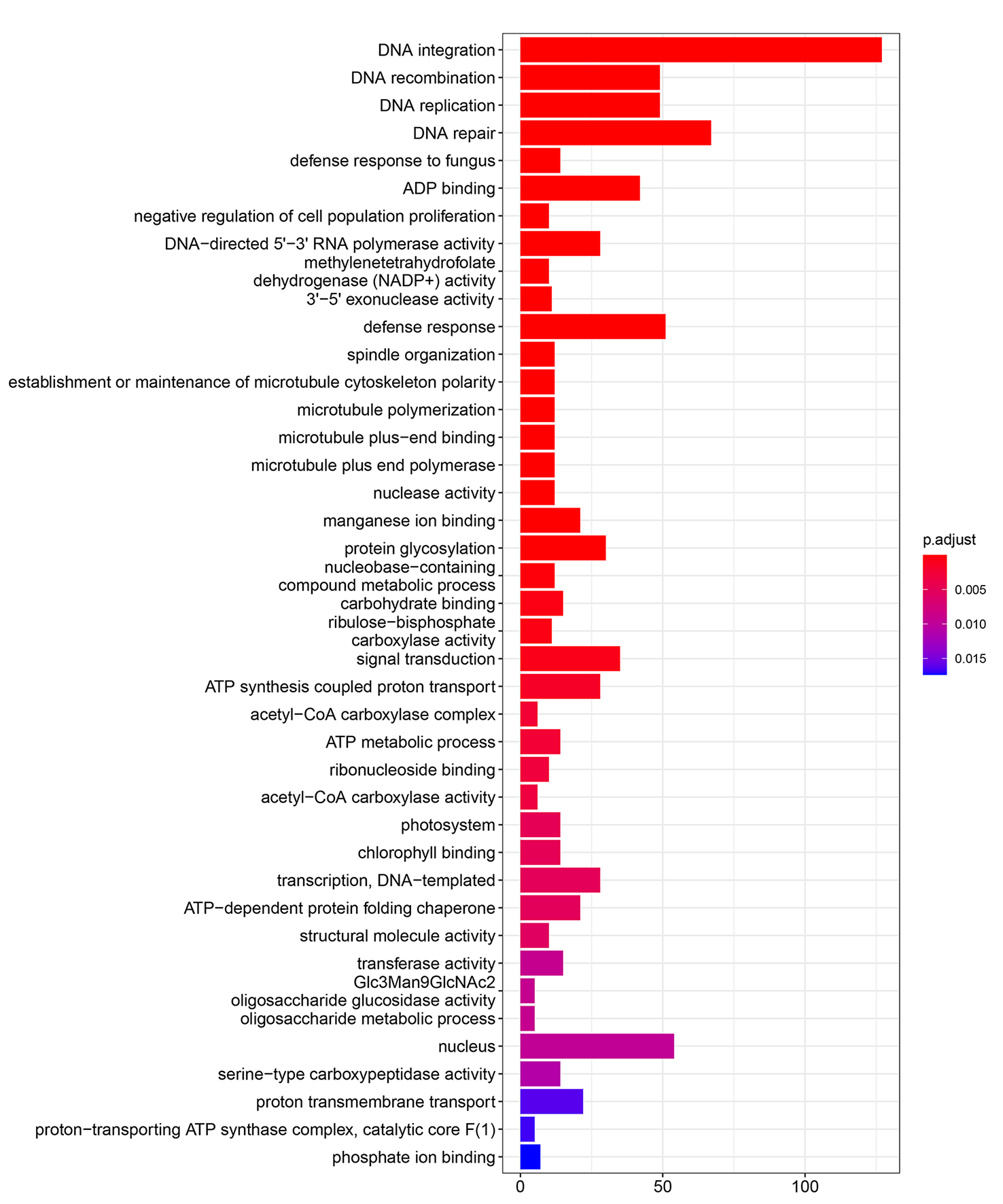

Supplement: Web_Material_uhad180 [file web_material_uhad180.zip › Fig. S3.tif]

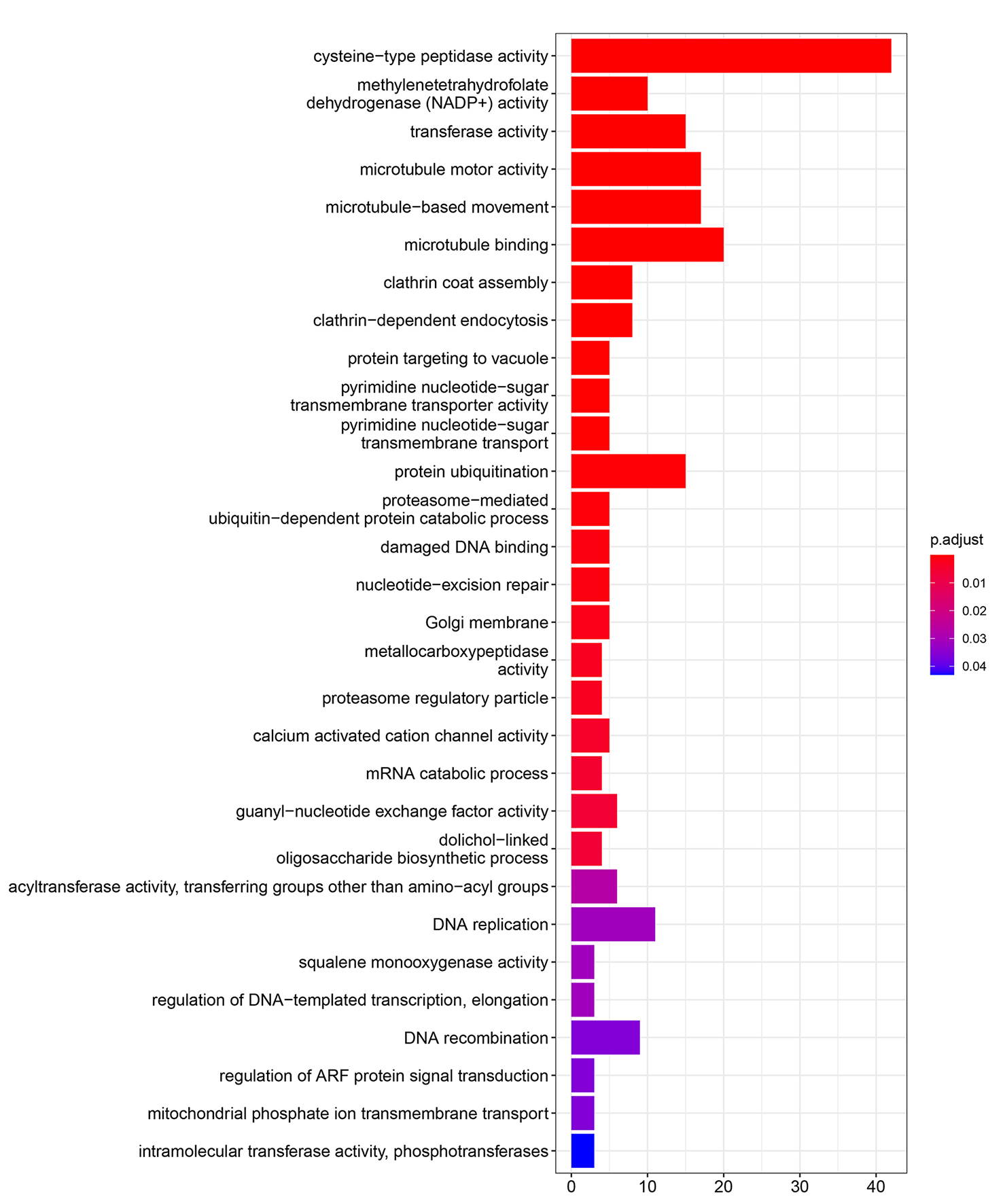

Supplement: Web_Material_uhad180 [file web_material_uhad180.zip › Fig. S4.tif]
