# Supplementary material for: Genome assembly of the snow lotus species Saussurea involucrata provides insights into acacetin and rutin biosynthesis and tolerance to an alpine environment
Source: Hortic Res. 2023 Sep 5;10(10):uhad180. doi: 10.1093/hr/uhad180 (PMC10599237; doi:10.1093/hr/uhad180)
Supplement: Web_Material_uhad180 [file web_material_uhad180.zip › Supplementary Data.revision.doc]

**Figure S1.** The syntenic pattern between genomic regions in *C. cardunculus* (artichoke) and *S. involucrata.*

**Figure S2.** Syntenic blocks (≥4 collinear genes) between *C. cardunculus* (artichoke) and *S. involucrata.*

**Figure S3.** Graphical results showing enriched GO terms for genes in gene families expanded in *S. involucrata*.

**Figure S4.** Graphical results showing enriched GO terms for genes in gene families specific to *S. involucrata*.

**Table S1.** Summary of repeat elements identified in the *Saussurea involucrata* genome assembly.

| **Class** | **Subclass** | **Number** | **Length (bp)** | **Percent** |
| --- | --- | --- | --- | --- |
| Retrotransposons |  | 586,263 | 1,004,960,602 | 40.99 % |
|  | LTR elements | 568,890 | 991,331,394 | 40.43 % |
|  | Gypsy | 255,125 | 354,969,081 | 14.48 % |
|  | Copia | 280,104 | 613,890,844 | 25.04 % |
|  | Pao | 17,718 | 6,432,862 | 0.26 % |
|  | Retroviral | 943 | 136,943 bp | 0.01 % |
|  | Non-LTR elements | 18,500 | 139,22,081 | 0.57 |
|  | LINEs | 17,373 | 13,629,208 | 0.56 % |
|  | Penelope | 1,127 | 292,873 | 0.01 % |
|  | SINEs | 0 | 0 | 0 |
| DNA transposons |  | 227,054 | 89,448,668 | 3.65 % |
|  | hobo-Activator | 22,994 | 7,744,846 | 0.32 % |
|  | Harbinger | 49077 | 17,440,713 | 0.71 % |
| Other repeats |  | 2,341,892 | 934,871,834 | 38.13% |
|  | Unkown | 1,978,406 | 889,108,071 | 36.26% |
|  | Satellites | 4,548 | 1281581 | 0.05 % |
|  | Simple repeats | 315,984 | 14,897,852 | 0.61 % |
|  | Low complexity | 36,270 | 1,936,211 | 0.08 % |
|  | Rolling-circles | 6,684 | 2,977,994 | 0.12 % |
| Total |  | 6,728,952 | 2029393735 | 82.77 % |

**Table S2.** Annotation statistics of predicted protein-coding genes for the *Saussurea involucrata* genome.

| **Category** | **Number** | **Percentage** |
| --- | --- | --- |
| Protein-coding genes | 44,486 | 100.00% |
| NCBI nr | 42,566 | 95.68% |
| Uniprot | 31,795 | 71.47% |
| Swiss-Prot | 31,802 | 71.49% |
| InterPro | 38,626 | 86.83% |
| GO | 23,284 | 52.34% |
| Total functionally annotated | 42,580 | 95.72% |
| Unannotated | 564 | 1.27% |

**Table S3.** *Saussurea involucrata* gene homologous to known cold resistance genes.

|  | **Gene in *S. involucrata*** | **Reference** |
| --- | --- | --- |
| transcription factor ICE1 | SnowLotus28074 | Wu CL, Lin LF, Hsu HC et al. *Saussurea involucrata* (Snow Lotus) ICE1 and ICE2 orthologues involved in regulating cold stress tolerance in transgenic *Arabidopsis*. Int J Mol Sci. 2021;22:10850. |
|  | SnowLotus03546 |  |
| calcium-dependent protein kinase 1 | SnowLotus15867 | Tian XH, Pang XB, Zhu JB et al. Effect of over-expression of *Saussurea involucrata* SikCDPK1 gene on improving cold  tolerance in transgenic tobacco. Acta Tabacaria Sinica. 2016;22:98-103. |
|  | SnowLotus36327 |  |
| dehydrin | SnowLotus36292 | Qiu H, Zhang L, Liu C *et al.* Cloning and characterization of a novel dehydrin gene, SiDhn2, from *Saussurea involucrata* Kar. et Kir. Plant Mol Biol. 2014;84:707-18. |
| [stearoyl-acyl-carrier-protein desaturase](https://www.so.com/link?m=bNMRCdeDfa57diFdoR/zwIVSElq7xkU10IZlLrt6AhLZCz6eMn4kdCd7L3p32hn7Ru3pewsMf2DKjw43+QBeU3AEc5cNpghD4lBa6zPckedUpxWTvZTLExM+36RqEQ4fCmBIAF2R80YEZbRUxtEXk+a+JNNOlKt41yITtg8dSoEc=) | SnowLotus35130 | Liu HL, Shen HT, Chen C *et al.* Identifification of a putative stearoyl acyl-carrier-protein desaturase gene from *Saussurea involucrata*. Biol. Plant. 2015;59:316–324. |
|  | SnowLotus36810 |  |
| fructose-bisphosphate aldolase | SnowLotus00644 | Mu J, Fu Y, Liu B *et al*. SiFBA5, a cold-responsive factor from *Saussurea involucrata* promotes cold resilience and biomass increase in transgenic tomato plants under cold stress. BMC Plant Biol. 2021;21:75. |
|  | SnowLotus18029 |  |
|  | SnowLotus25464 |  |
|  | SnowLotus31382 |  |
| late embryogenesis abundant protein | SnowLotus19835 | Kong H, Xia W, Hou M et al. Cloning and function analysis of a *Saussurea involucrata* LEA4 gene. Front Plant Sci. 2022;13:957133. |
|  | SnowLotus26899 |  |
| cold-regulated 413 plasma membrane protein | SnowLotus11713 | Guo X, Zhang L, Dong G et al. A novel cold-regulated protein isolated from *Saussurea involucrata* confers cold and drought tolerance in transgenic tobacco (Nicotiana tabacum). Plant Sci. 2019;289:110246. |
|  | SnowLotus34879 |  |
